# Genetic Code Expansion: Another Solution to Codon Assignments

**DOI:** 10.3390/ijms24010361

**Published:** 2022-12-26

**Authors:** Kensaku Sakamoto

**Affiliations:** Laboratory for Nonnatural Amino Acid Technology, RIKEN Center for Biosystems Dynamics Research, 1-7-22 Suehiro-cho, Tsurumi-ku, Yokohama 230-0045, Japan; kensaku.sakamoto@riken.jp

This Special Issue is intended to highlight recent advances in genetic code expansion, particularly the site-specific incorporation of noncanonical amino acids (ncAAs) into proteins. This research field has two different targets: building the decoding systems composed of ncAAs, tRNAs, and aminoacyl-tRNA synthetases (aaRSs), each orthogonal to their natural counterparts in cells; and applications of these systems to protein science and engineering, molecular biology, pharmaceutical developments and the materials industry. Chromosome engineering has also become part of genetic code expansion since researchers succeeded in fully-fledged codon reassignments [1,2,3,4]. If one of the 64 codons is erased from the genome, the meaning of this codon becomes blank and can be redefined with any ncAA, provided that its specific tRNA/aaRS pair is expressed in cells. When multiple codons thus go blank, the challenge is to engineer enough numbers of the orthogonal tRNA/aaRS systems (OTSs) for filling the blank codons with new meanings.

A dozen orthogonal OTSs have been derived from the naturally occurring tRNA/aaRS pairs by exploiting the fact that particular nucleotides of tRNA (identity elements) define the specific interaction between tRNA and aaRS, and vary among organisms from different kingdoms [5]. Still, genetic code expansion has been focused on only two OTSs based on tyrosyl-tRNA synthetase (TyrRS) and pyrrolysyl-tRNA synthetase (PylRS), largely because these two synthetases recognize most parts of the available ncAA collection. Recently, researchers succeeded in engineering mutually orthogonal PylRS/tRNA^Pyl^ systems, which are useful for incorporating distinct ncAAs simultaneously into proteins [6,7]. Given the currently available ncAAs, the combination between the tyrosine and pyrrolysine OTSs is most promising for encoding multiple ncAAs in one genetic code. Although, it is also feasible to co-use other synthetases together with these OTSs to increase the variety of ncAAs in proteins [8]. On the other hand, changing the tRNA specificity of aaRS to create more OTSs is still challenging. Although tRNA identity elements occur in confined parts of the molecule, dozens of amino acid residues of aaRS interact with tRNA nucleotides to distinguish tRNA tertiary structure, as well as specific identity elements. Engineered variations in tRNA structure might help develop artificial aaRS/tRNA pairs, although difficulties in rearranging tRNA structure hamper this direction. Two studies included in this Special Issue are attempts to create new tRNA formats that guarantee orthogonality and high translational efficiency [9,10]. 

Changing the amino acid specificity of aaRSs in favor of ncAAs has been another central issue in genetic code expansion. Significantly fewer residues of aaRS are in contact with a substrate amino acid than with tRNA, and have been the subject of mutagenesis for changing the substrate specificity. Constant efforts have allowed the addition of hundreds of ncAAs to the genetically encoded repertoire. The aaRS variants thus engineered are applicable to not only site-specific ncAA incorporation, but also to residue-specific incorporation in cell-based and cell-free systems. Two main issues concerning amino acid specificity are yet to be overcome. First, there is a serious limitation on the variety of available ncAAs, reflecting the dominant use of TyrRS and PylRS; most of the genetically encoded ncAAs are either tyrosine or lysine derivatives. A clear trend that has emerged involves engineering PylRS toward various amino acids, including tyrosine derivatives. Koch et al. reported that a series of small aliphatic amino acids were incorporated into proteins by engineered PylRSs, further illuminating the plasticity of the substrate recognition by this synthetase [11]. Secondly, low incorporation efficiency due to the poor ncAA recognition rates of engineered synthetases reduces the utility of many ncAAs. A larger-scale remodeling of the amino acid binding pocket may be the answer. Substrate recognition involves more than twenty residues of aaRS, which are those in direct contact with the substrate and the second-layer residues supporting the configurations of the first ones. The number of possible permutations of mutagenesis is quite large, and surpasses experimental capacity. Rapidly developing computational tools would be applicable to tackling this burden of number [12]. Structural knowledge of aaRS variants is accumulating, and helpful for engineering amino acid specificity. By X-ray crystallography, Hosaka et al. revealed two distinct recognition modes for the common nitrobenzene moiety of two different caged tyrosine derivatives; the same chemical group protrudes from the tyrosine ring in opposite directions, and thus different sets of mutations are required to recognize the group [13]. Structural knowledge, alongside computational approaches and experimental selection schemes that have steadily been sophisticated, will facilitate the remodeling of the binding pockets [14,15,16].

Translation machinery is composed of many cellular components, including ribosomes and elongation factors. The efficiency of protein biosynthesis with ncAA is the outcome of the whole machinery, and the poor activities of aaRS variants toward ncAAs may not be solely responsible for inefficient incorporation. Katoh and Suga reviewed recent studies on the alterations of the ribosome EF-Tu and of tRNA for enhancing the consecutive incorporation of d- and β-amino acids into proteins [17]. The biosynthesis of proteins containing ncAAs in a row is still problematic for many ncAAs, hampering applications that demand it. The difficulty of altering the translational components comes partly from the adverse effects that modified components can cause in cells. Cell-free synthesis has been a powerful method for incorporating ncAAs into proteins, and can safely be used for engineering variants of the essential components. Continuous efforts have been aimed at improving the cell-free system, which enables the incorporation of ncAAs into proteins that are difficult to synthesize in vivo [18].

This Special Issue includes five articles and reviews in the application domain. The zebrafish genetic code was expanded to include reactive amino acids useful for photo-crosslinking and chemical conjugation [19], while transgenic silkworms produced azido-functionalized silk fiber on a gram-order scale [20]. Braun et al. gave an overview of the site-directed spin-labeling of proteins for studying structural dynamics and protein–protein interactions [21]. Whereas most applications involve the incorporation of ncAAs into proteins, Kato’s direction is unique, using ncAAs for regulating gene expression [22] and controlling plasmid maintenance and exchange [23]. Codon reassignments allow ncAAs to be incorporated at multiple specific sites in proteins, constitute integral parts of the protein structure and function like standard amino acids. We now have a few blank codons that can be used to encode different ncAAs in a single genetic code. This capability calls for new applications in protein design, biomaterial development and biologics engineering.
